# The Influence of Cesarean Section on the Composition and Development of Gut Microbiota During the First 3 Months of Life

**DOI:** 10.3389/fmicb.2021.691312

**Published:** 2021-08-18

**Authors:** Gao Long, Yuting Hu, Enfu Tao, Bo Chen, Xiaoli Shu, Wei Zheng, Mizu Jiang

**Affiliations:** Department of Gastroenterology, Children’s Hospital, Zhejiang University School of Medicine, National Clinical Research Center for Child Health, National Children’s Regional Medical Center, Zhejiang, China

**Keywords:** gut microbiota, cesarean section, Firmicutes/Bacteroidetes ratio, development, obesity

## Abstract

The intestinal microbiota has emerged as a critical regulator of growth and development in the early postnatal period of life. Cesarean section (CS) delivery is one of the strongest disrupting factors of the normal colonization process and has been reported as a risk factor for disorders in later life. In this study, we dynamically and longitudinally evaluated the impact of CS on the initial colonization pattern and development of gut microbiota by 16 healthy Chinese infants with fecal samples collected at 9 time points (day 5, day 8, day 11, week 2, week 4, week 6, week 7, month 2, and month 3) during the first 3 months of life. The V3–V4 regions of *16S rRNA* gene were analyzed by Illumina sequencing. In comparison with vaginally delivered (VD) infants, infants born by CS showed decreased relative abundance of *Bacteroides* and *Parabacteroides* and enrichment of *Clostridium_sensu_stricto_1*, *Enterococcus*, *Klebsiella*, *Clostridioides*, and *Veillonella*. Most interestingly, Firmicutes/Bacteroidetes ratio was found to be significantly higher in the CS group than in the VD group from day 5 until month 3. Besides, the results of microbial functions showed that the VD group harbored significantly higher levels of functional genes in vitamin B6 metabolism at day 5, day 8, week 2, week 4, week 6, week 7, month 2, and month 3 and taurine and hypotaurine metabolism at day 5, while the phosphotransferase system and starch and sucrose metabolism involved functional genes were plentiful in the CS group at day 11, week 2, week 4, week 6, week 7, and month 2 and at week 2, week 7, and month 2, respectively. Our results establish a new evidence that CS affected the composition and development of gut microbiota in the first 3 months and provide a novel insight into strategies for CS-related disorders in later life.

## Introduction

Accumulating evidence has shown that the gut microbiota plays a fundamental role in the health and disease by assisting in the synthesis and absorption of nutrients, strengthening gut integrity, protecting against enteropathogens, modulating the immune system, and exerting control over the gut–brain axis (Bäumler and Sperandio, 2016; Adak and Khan, 2019; Ahern and Maloy, 2020). The gastrointestinal tract contains 10^14^ microorganisms, which encompasses 10 times more bacterial cells than the total number of human cells (Thursby and Juge, 2017). The gut is of particular relevance to human health, as it contains the majority and most diverse set of human commensal bacteria (Savage, 1977). In the gut microbiota, bacteria are an essential part, and they are also the most commonly studied. The 16S rRNA high-throughput sequencing has become a popular technology for detecting intestinal flora in recent years due to its characteristics of fast sequencing, detailed results, and high accuracy (Fouhy et al., 2012). It is well known that the dynamic infant gut microbiota symbiosis is established from birth and developed rapidly in the first 3 years of life, particularly during infancy (Robertson et al., 2019; Shao et al., 2019). The early postnatal period of life is a vital stage for growth and development. The intestinal microbiota has emerged as a critical regulator of growth and development in the early postnatal period of life (Lee et al., 2021). The intestinal microbiota is deemed to coincide with the development of our body systems (Cowan et al., 2020; Wilkins and Reimer, 2021). Accordingly, perturbations influenced by any detrimental factors in early life may cause adversely long-lasting consequences for host health (Klancic and Reimer, 2020), ranging from gastrointestinal diseases, such as inflammatory bowel disease (Zuo et al., 2018) and irritable bowel syndrome (Rincel and Darnaudéry, 2020), immune reactivity, and immunopathology (Al Nabhani and Eberl, 2020) to allergies, including asthma, food allergy, and atopic dermatitis (Zhuang et al., 2019; Rachid et al., 2021; Renz and Skevaki, 2021).

The maturation of gut microbiota is strongly influenced by both internal host properties and external factors, such as ethnicity, gestational age, delivery mode, feeding pattern, and antibiotic usage, of which cesarean section (CS) is a crucial one (Heiss and Olofsson, 2019; Kim et al., 2019; Shao et al., 2019). It is reported that CS delivery is one of the strongest disrupting factors of the normal colonization process (Korpela et al., 2020). The microbial population of neonates born vaginally resembles the maternal vagina and perianal microbes, while CS-delivered neonates predominantly acquire bacteria derived from the maternal skin and the surrounding environment (Dominguez-Bello et al., 2010). Maternal microbiota exerts a significant effect on the neonatal microbiome and contributes to regulating the development of offspring immunity, metabolism, brain function, and behavior (Jašarević and Bale, 2019; García-Mantrana et al., 2020). These advantage effects of maternal microbiota can be vertically transmitted to offspring through vaginal delivery (Mueller et al., 2016). Despite CS can be a life-saving intervention when medically indicated, this procedure can lead to short-term and long-term health effects for children with the absence of transmission from maternal microbiota to offspring (Sandall et al., 2018). There is growing evidence that CS delivery is a risk factor for the onset of several clinical diseases later in life including asthma (Black et al., 2015), atopic disease (Seo et al., 2015), allergies (Sandall et al., 2018), and obesity (Kuhle et al., 2015). Furthermore, CS delivery has been reported to increase the risk of respiratory infections in later childhood (Unger and Bogaert, 2017) and impair cognitive capabilities in school-age children (Polidano et al., 2017). The correlation between CS delivery and these increased risks may be interpreted by the subsequent disturbance in immune response regulation after the absence of contact with maternal vaginal microbes at birth (Neu and Rushing, 2011).

Overall, the first 3 months of early life is the first peak of growth and development. Given the effects of CS delivery on the developing microbial colonization process in infants may pose risks for morbidity in later life, it is indispensable to characterize early gut microbiota profiles based on the influence of delivery mode for elucidating the mechanisms underlying their relationship with affected diseases. However, few studies have focused on the influence of CS on the dynamic of gut microbiota, particularly on this critical period. Therefore, we longitudinally collected fecal samples from a cohort of 16 healthy full-term Chinese infants at 9 time points throughout the first 3 months of life and aimed to identify the effects of CS delivery on the composition of gut microbiota by high-throughput sequencing of the V3–V4 regions of *16S rRNA* gene in such period. To predict the functional potential of intestinal flora and point out the direction for the subsequent research, the PICRUSt software was used based on its high accuracy (>90% in most cases) (Douglas et al., 2018).

## Materials and Methods

### Study Design and Participants

The study was approved by the Ethics Committee of the Children’s Hospital of Zhejiang University School of Medicine. Written informed consent was obtained from mothers before collecting samples. The longitudinal cohort study was conducted between November 6, 2017 and December 5, 2018. Healthy infant volunteers who met the following criteria were recruited in our study: (1) whose mothers worked and were residents in the city of Hangzhou, China and (2) full-term healthy infants born between gestational age of 37 and 41 weeks; any subjects containing one of the following conditions were excluded: (1) infants born from mothers who had any infection during the third trimester of pregnancy and chronic diseases, such as diabetes, hypertensive disorders, or autoimmune disease and (2) infants who presented any abnormal conditions, such as neonatal asphyxia, hyperbilirubinemia, and any other conditions that needed extensive care. Parents were educated for fecal sampling and storage. Fecal samples were collected by parents at 9 time points during the first 3 months of life (day 5, day 8, day 11, week 2, week 4, week 6, week 7, month 2, and month 3 after birth). Clinical data regarding both mothers and infants including maternal age, gravidity and parity history, mode of delivery, sex, gestational age, birth weight, and other factors, such as family smoking and pet keeping, were recorded. A total of 16 infant volunteers were recruited in the longitudinal cohort study, including 6 of vaginally delivered (VD) and 10 of CS. 123 fecal samples were collected at 9 time points in the first 3 months of life.

### Fecal Sample Collection

Fecal samples were collected into sterile containers by parents and immediately frozen in a −20°C refrigerator. Samples were then transported to the laboratory using dry ice by investigators and were frozen at −80°C for subsequent experimentation.

### Genomic DNA Extraction

Genomic DNA was isolated from fecal samples using the QIAmp Fast DNA Stool Mini Kit (51604; Qiagen, Valencia, CA, United States) in accordance with the manufacturer’s instructions. DNA was eluted with 50 μl elution buffer and stored at −80°C before use. The DNA extract was checked on 1% agarose gel, and DNA concentration and purity were assessed by NanoDrop 2000 UV–vis spectrophotometer (Thermo Scientific, Wilmington, DE, United States).

### Sequencing of *16S rRNA* Gene

The sequencing of *16S rRNA* gene was performed as previously reported method (Zheng et al., 2019, 2020). Concretely, the V3–V4 hypervariable regions of the bacteria *16S rRNA* gene were amplified with barcode-indexed primers 338F (5′-ACTCCTACGGGAGGCAGCAG-3′) and 806R (5′-GGACTACHVGGGTWTCTAAT-3′) by an ABI GeneAmp^®^ 9700 PCR thermocycler (ABI, Foster City, CA, United States). The PCR amplification mixture contained: 4 μl 5 × *TransStart* FastPfu Buffer, 2 μl 2.5 mM dNTPs, 0.8 μl forward primer (5 μM), 0.8 μl reverse primer (5 μM), 0.4 μl *TransStart* FastPfu DNA Polymerase, 0.2 μl BSA, 10 ng template DNA, and finally ddH_2_O up to 20 μl. The PCR conditions for amplification were as follows: initial denaturation for 3 min at 95°C, followed by 27 cycles of denaturation at 95°C for 30 s, annealing at 55°C for 30 s and extension at 72°C for 45 s, and single extension at 72°C for 10 min, and end at 10°C. PCR reactions were performed in triplicate. PCR products were extracted from 2% agarose gel, purified by the AxyPrep DNA Gel Extraction Kit (Axygen Biosciences, Union City, CA, United States), and quantified using Quantus^TM^ Fluorometer (Promega, Madison, WI, United States) according to the manufacturer’s instructions. Purified amplicons were pooled in equimolar and paired-end sequenced on an Illumina MiSeq platform (Illumina, San Diego, CA, United States) according to the standard protocols by Majorbio Bio-Pharm Technology Co., Ltd. (Shanghai, China).

### Sequence Data Processing and Statistical Analysis

The protocol of sequence data processing was similar to the previously published study (Zheng et al., 2020). Briefly, the raw *16S rRNA* gene sequencing reads were demultiplexed, quality-filtered by Trimmomatic (Bolger et al., 2014), and merged by FLASH (Magoc̀ and Salzberg, 2011) with the following criteria: (i) the 300 bp reads were truncated at any site receiving an average quality score of < 20 over a 50-bp sliding window, and the truncated reads shorter than 50 bp were discarded; reads containing ambiguous characters were also discarded; (ii) only overlapping sequences longer than 10 bp were assembled according to their overlapped sequence. The maximum mismatch ratio of the overlap region was 0.2. Reads that could not be assembled were discarded; (iii) samples were distinguished according to the barcode and primers, and the sequence direction was adjusted. The allowed maximum barcode mismatch number was 0, and the primer mismatch number was 2. Operational taxonomic units (OTUs) with 97% similarity cutoff were clustered using UPARSE (Edgar, 2013) (version 7.1,^1^), and chimeric sequences were identified and removed. To minimize the effects of sequencing depth on alpha and beta diversity measures, the number of reads from each sample was rarefied to 29,101, which still yielded an average Good’s coverage of 99.60%. The taxonomy of each OTU representative sequence was analyzed by RDP Classifier (Wang et al., 2007)^2^ against the 16S rRNA database (Silva 132) using the confidence threshold of 0.7. Taxonomic relative abundance profiles at taxa levels (domain, kingdom, phylum, class, order, family, and genus) were generated based on OTU annotation. Principal coordinates analysis (PCoA) was performed based on Weighted Unifrac distance to visualize differences in bacterial structure of infants under delivery modes and sampling time points. Before Unifrac analysis, the phylogenetic tree was constructed with representative OTU sequences by FastTree version 2.1.3 and MUSCLE (multiple sequence comparison by log-expectation). Differences in microbial composition and abundance between groups of PCoA were tested using the analysis of similarities (ANOSIM) on the beta diversity matrix. Generalized Linear Models with participant identity as a random effect were used to determine the variation tendency of gut microbial abundance with age. And the Wilcoxon rank-sum test was used to determine the significant differences between groups at both phylum and genus levels for abundance of gut microbiota, and in the microbial functional gene composition. The microbiota enterotype analysis was performed based on R (version 3.3.1) combined with Jensen–Shannon divergence (JSD) distance to evaluate the distinct growth pattern of gut microbiota according to delivery mode. To predict the microbial functions, functional prediction and annotations were conducted using the Phylogenetic Investigation of Communities by Reconstruction of Unobserved States (PICRUSt) (Douglas et al., 2018). The distribution of data, such as infant information (gestational age and birth weight), maternal information (age, gravidity, parity, pre-pregnancy weight, and pregnancy weight), and Firmicutes/Bacteroidetes (F/B) ratio, were analyzed by Kolmogorov–Smirnov test. Thereinto, normally distributed data (gestational age, birth weight, maternal age, gravidity, parity, pre-pregnancy weight, and pregnancy weight) were expressed as mean ± SD, and data between two groups were compared using the Student’s *t*-test. Non-normally distributed data (F/B ratio) were presented as median (minimum, maximum) unless specifically highlighted, and data between two groups were compared using the Mann–Whitney *U* test. Countable data, such as infant sex and other information (family smoking, pet keeping), were presented as number (%) and tested using chi-square test.

## Results

### General Characteristics of the Subjects

**FIGURE 1:**
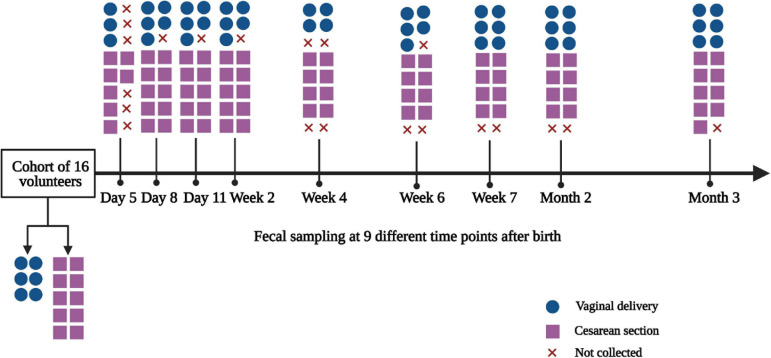
Flow diagram of the study. The diagram showed the fecal samples collected at 9 time points. x, fecal samples were not collected by parents as the infants had no defecation.

The flow diagram of the study is generalized in Figure 1. The general characteristics of the study population according to delivery mode are summarized in Table 1. There were no significant differences in the baseline characteristics between VD and CS-delivered infants, such as infant information (gestational age and birth weight) and maternal information (age, gravidity, parity, pre-pregnancy weight, and pregnancy weight) (Student’s *t*-test, all *p* > 0.05), as well as infant information (sex) and other information (family smoking and keeping pets) (chi-square test, all *p* > 0.05).

**TABLE 1:** General characteristics of infants enrolled in the study according to delivery mode.

**Variable**	**VD (*n* = 6)**	**CS (*n* = 10)**	***x*^2^/*F***	***p***
Infant information	6 (37.50)	10 (62.50)		
Male, *n* (%)	3 (42.86)	4 (57.14)	0.15	0.55
Female, *n* (%)	3 (33.33)	6 (66.67)		
Gestational age, week	39.17 ± 1.17	38.60 ± 0.97	0.10	0.31
Birthweight, g	3,368.33 ± 337.40	3,246.00 ± 436.25	1.44	0.57
Maternal information	6 (37.5)	10 (62.5)		
Age, year	30.00 ± 3.16	33.20 ± 5.25	0.98	0.20
Gravidity	1.67 ± 0.82	2.00 ± 1.25	1.25	0.57
Parity	1.50 ± 0.55	1.50 ± 0.53	–	1.0
Pre-pregnancy weight, kg	52.33 ± 5.35	52.85 ± 5.27	0.27	0.85
Pregnancy weight, kg	64.67 ± 6.97	68.35 ± 5.03	2.67	0.24
Other information				
Family smoking, n (%)	0 (0)	0 (0)	–	–
Pet keeping, n (%)	0 (0)	0 (0)	–	–

*VD, vaginal delivery; CS, cesarean section. Countable data between two groups were tested using chi-square test; continuous variables between two groups were compared using the Student’s *t*-test.*

### Comparison of Gut Microbial Diversity

**FIGURE 2:**
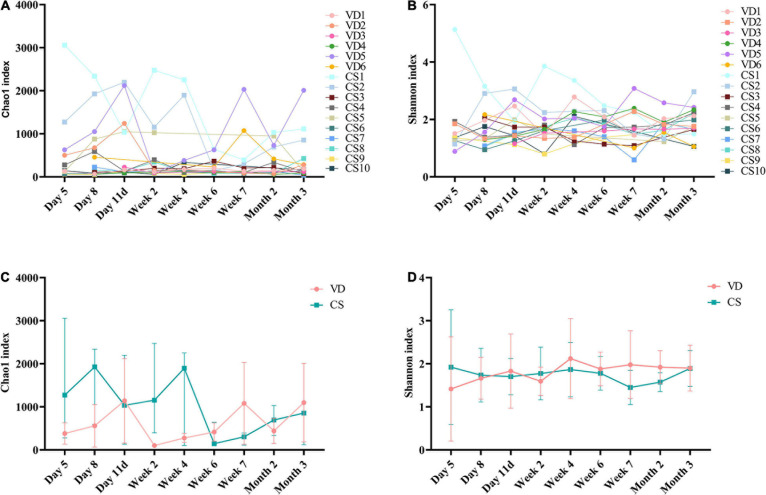
Alpha diversity dynamic of the gut microbiota based on 9 time points between infants born with vaginal delivery and born with cesarean section. **(A)** Chao1 index; **(B)** Shannon index; **(C)** the comparison of Chao1 index between VD and CS at 9 time points (Mann–Whitney *U* test, all *p* > 0.05); **(D)** the comparison of Shannon index between VD and CS at 9 time points (Mann–Whitney *U* test, all *p* > 0.05). The results were reported as median values (minimum–maximum). VD, vaginal delivery; CS, cesarean section.

The rarefaction curves showed that curves have reached a flat level, indicating that the sequencing depth was sufficient (Supplementary Figure 1). The Chao1 (Figure 2A) and Shannon (Figure 2B) indexes, which represent microbial alpha diversity and microbiota richness, showed non-coordinated dynamics for all individuals in the infants both born with VD and born with CS. The results showed that there was no statistical difference both in the Chao1 (Mann–Whitney *U* test, all *p* > 0.05; Figure 2C) and in the Shannon index (Mann–Whitney *U* test, all *p* > 0.05; Figure 2D) at 9 time points between the two groups.

### Impact of CS on Temporal Changes of Gut Microbial Composition During the First 3 Months of Life

**FIGURE 3:**
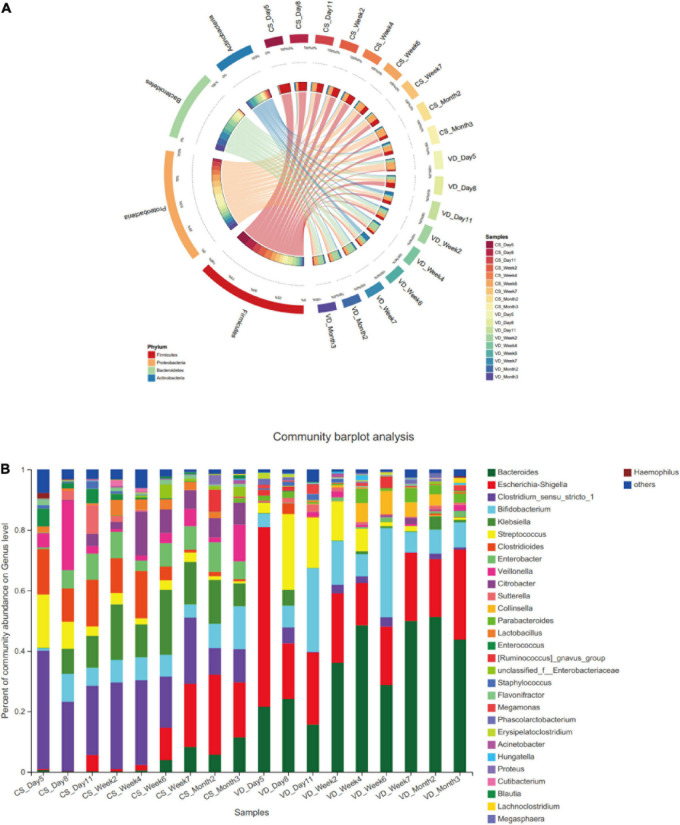
Dynamic development of microbiota in the vaginal delivery and cesarean section groups. **(A)** Dynamic development of microbiota in phylum level; **(B)** dynamic development of microbiota in genus level; CS, cesarean section; VD, vaginal delivery.

In two delivery mode groups, we determined the most predominant 4 phyla for the first 3 months of life: Firmicutes, Proteobacteria, Bacteroidetes, and Actinobacteria (Figure 3A). Despite the 4 dominant phyla were present in both groups, we observed a clear distinction of temporal changes in their proportions at the phylum level between the two groups. Within the CS group, Firmicutes was observed as the most dominant phylum from day 5 to week 4 after birth. The relative abundance of Firmicutes gradually decreased from day 5 to month 3 after birth (Supplementary Table 1, Generalized Linear Models, *p* = 0.000). In contrast, the prevalence of Proteobacteria continuously increased from day 5 to month 3 after birth (Generalized Linear Models, *p* = 0.006) and dominated from week 6 to month 3 in the CS group. Additionally, Bacteroidetes had a lower abundance from day 5 to week 4 and then increased gradually from week 6 to month 3 in the CS group. Whereas in the VD group, the predominant phyla from day 5 to day 11 were Proteobacteria and Firmicutes. The relative proportion of Firmicutes significantly decreased from day 5 to month 3 (Generalized Linear Models, *p* = 0.021). At the genus level, several genera demonstrated differences in variation tendency between two delivery mode groups (Figure 3B). For instance, the relative abundance of *Escherichia*–*Shigella* displayed an increasing trend from day 5 to month 3 in CS infants (Supplementary Table 2, Generalized Linear Models, *p* = 0.001). *Clostridioides* and *Streptococcus* showed the decreasing tendency in abundance from day 5 to month 3 in the CS group (Generalized Linear Models, *p* < 0.01), whereas genera in the VD group did not show obvious variation tendency.

### Impact of CS on Temporal Change of F/B Ratio

**FIGURE 4:**
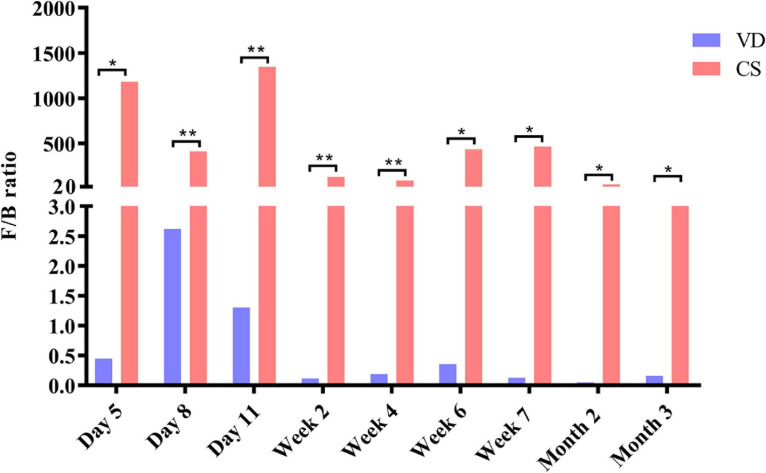
Cesarean section significantly increased Firmicutes/Bacteroidetes ratio in the first 3 months of life. Data were representative with median. F/B ratio: Firmicutes/Bacteroidetes ratio; CS: cesarean section; VD: vaginal delivery. *p < 0.05; **p < 0.01 (Mann–Whitney *U* test).

Cesarean section delivery significantly affected F/B ratio from day 5 to month 3 compared with VD delivery (Figure 4, Mann–Whitney *U* test, all *p* < 0.05). F/B ratio was significantly higher in the CS group than in the VD group at every time point (Figure 4, Mann–Whitney *U* test, all *p* < 0.05).

### Comparison of the Gut Microbiota Composition by the Mode of Delivery

**FIGURE 5:**
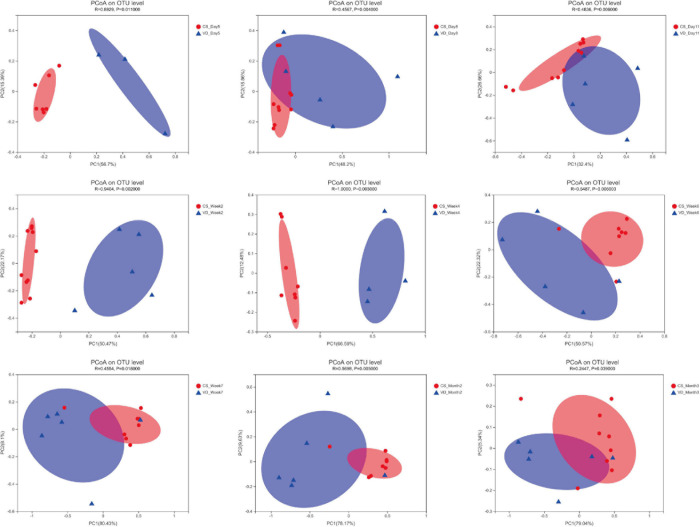
Principal coordinates analysis of the infant’s gut microbiota among different time points based on delivery mode. *R* and *p* were calculated using ANOSIM.

**FIGURE 6:**
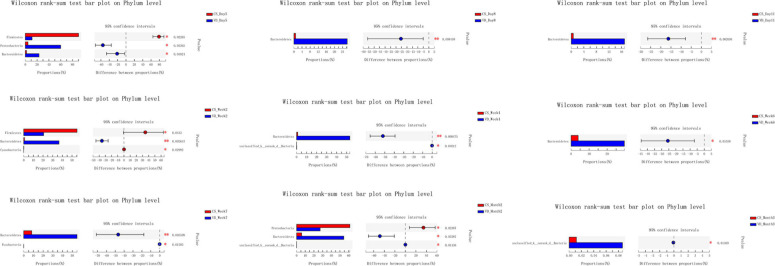
Cesarean section significantly affected the development of gut microbiota at the phylum level in the first 3 months of life. *p < 0.05; **p < 0.01 (Wilcoxon rank-sum test).

**FIGURE 7:**
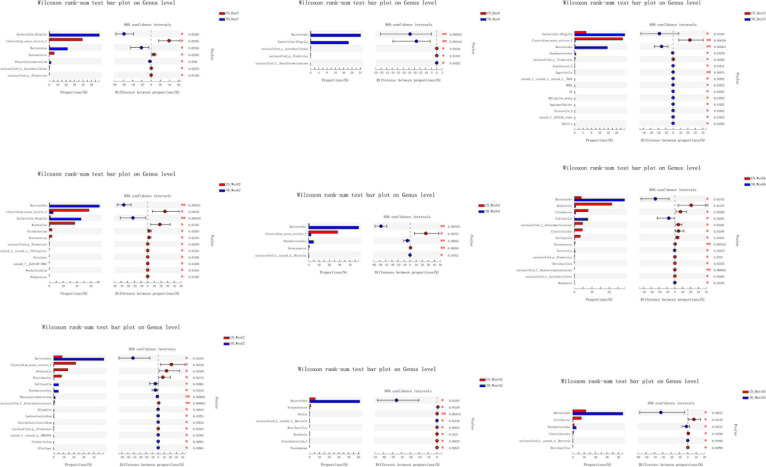
Cesarean section significantly affected the development of gut microbiota at the genus level in the first 3 months of life. *p < 0.05; **p < 0.01 (Wilcoxon rank-sum test).

The overall difference in microbiota community between two groups (beta diversity) was compared using PCoA based on Weighted Unifrac distance combined with ANOSIM, which distinctly revealed the effect of delivery mode on the gut microbiota at 9 time points (Figure 5, ANOSIM, all *p* < 0.05). The PCoA plot showed differences in bacterial phylogenetic structure between the CS and VD groups at every time point during the first 3 months in life. And the ANOSIM results revealed that such differences between two groups at every time point were statistically significant (Figure 5, ANOSIM, all *p* < 0.05). Thereafter, the significance test of the difference between groups was conducted by Wilcoxon rank-sum test at both phylum and genus levels. Concerning the phylum level, Firmicutes was found to be significantly more abundant in the CS group than in the VD group at day 5 and week 2, while Bacteroidetes exhibited significant superiority in the VD group at day 5, day 8, day 11, week 2, week 4, week 6, week 7, and month 2 (Figure 6, Wilcoxon rank-sum test, *p* < 0.05). Concerning the genus level, the microbiota of the VD group was characterized by significantly high levels of *Escherichia*–*Shigella* at day 5, day 8, day 11, and week 2 and persistent high levels of *Bacteroides* at 9 time points in comparison with the CS group. Besides, *Phascolarctobacterium* and *Parabacteroides* were also significantly more enriched in VD infants at day 5 and week 7 and at day 11, week 4, week 7, and month 3, respectively. We also observed that the VD group had a higher relative proportion of *Collinsella* at week 6 and week 7 than the CS group. As for infants delivered by CS, *Clostridium_sensu_stricto_1* (at day 5, day 11, week 2, week 4, and week 7), *Enterococcus* (at day 5, week 2, week 4, and week 6), *Klebsiella* (week 2, week 6, and week 7), *Clostridioides* (week 6, month 3), *Citrobacter* (week 6, month 3), *Streptococcus* (month 2), *Veillonella* (week 6), and *Enterobacter* (week 7) displayed significantly higher relative abundance (Figure 7, Wilcoxon rank-sum test, *p* < 0.05).

### The Microbiota Enterotype Analysis Based on Delivery Mode

**FIGURE 8:**
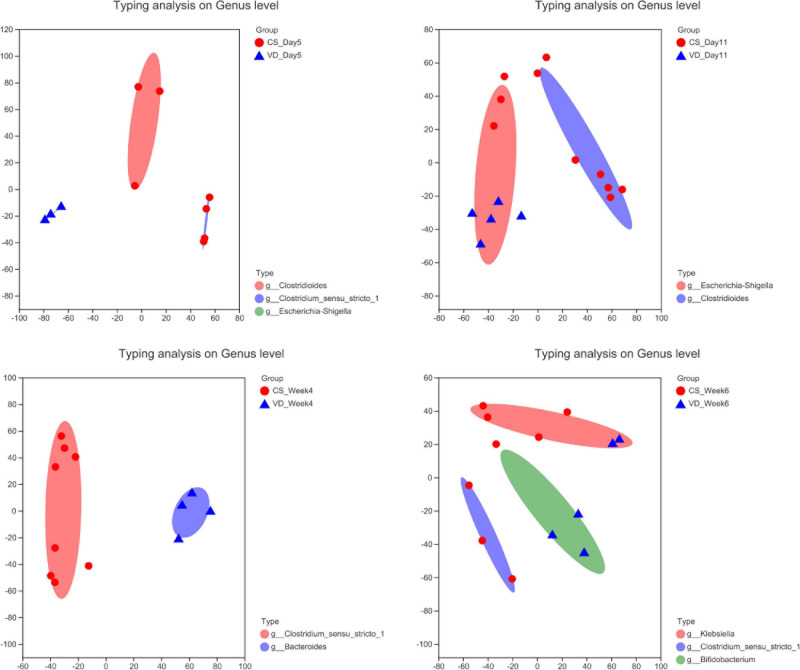
Enterotype analysis of gut microbiota based on delivery mode at different stages.

To evaluate the distinct growth pattern of gut microbiota according to delivery mode, the microbiota enterotype was analyzed at specific time points. As shown in Figure 8, enterotypes mainly represented by *Clostridioides* and *Clostridium_sensu_stricto_1* were predominant in the CSgroup at day 5, while the VD group possessed enterotype dominated by *Escherichia*–*Shigella*. In addition, *Clostridioides* and *Escherichia*–*Shigella*-dominated enterotypes were abundant in the CS and VD groups at day 11, respectively. Furthermore, we found the enrichment of *Clostridium_sensu_stricto_1* and *Bacteroides*-represented enterotypes at week 4 in the CS and VD groups, respectively. Besides, the CS group was predominated by *Klebsiella* and *Clostridium_sensu_stricto_1*-represented enterotypes, whereas *Bifidobacterium*-dominated enterotype was pronounced in VD infants at week 6.

### Comparison of Microbial Functions According to the Delivery Mode

Changes in microbial functions by different delivery modes using PICRUSt were predicted. We found significant differences in the functional gene composition in the metabolic pathway between two delivery mode groups (Supplementary Figures 2–4, Wilcoxon rank-sum test, *p* < 0.05). The results showed that the VD group harbored significantly higher levels of functional genes in vitamin B6 metabolism at day 5, day 8, week 2, week 4, week 6, week 7, month 2, and month 3 and taurine and hypotaurine metabolism at day 5 (Supplementary Figures 2–4, Wilcoxon rank-sum test, *p* < 0.05), whereas the phosphotransferase system (PTS) and starch and sucrose metabolism involved functional genes were plentiful in the CS group at day 11, week 2, week 4, week 6, week 7, and month 2 and at week 2, week 7, and month 2, respectively (Supplementary Figures 2–4, Wilcoxon rank-sum test, *p* < 0.05).

## Discussion

In this study, we explored the effects of CS on the development of gut microbiota in healthy Chinese infants by longitudinally collected fecal samples analyzed by *16S rRNA* gene sequencing during the first 3 months of life. Our results demonstrated the significant perturbation of the early colonization of gut microbiota caused by the CS compared with VD. Concordant with previous reports, we found that infants delivered by CS exhibited a decreased colonization of Bacteroidetes and an increased abundance of the Firmicute phylum (Jakobsson et al., 2014; Rutayisire et al., 2016; Yang R. et al., 2019), which might be interpreted by the lack of contact with maternal vaginal microbiota during childbirth (Rutayisire et al., 2016). Of particular importance, the colonization with *Bacteroides* was evidently delayed in infants born by CS, which was in line with previous findings (Hesla et al., 2014; Lee et al., 2016; Rutayisire et al., 2016; Kim et al., 2019; Shao et al., 2019). *Bacteroides* genus was thought to be capable of activating T-cell-dependent immune responses, thereby modulating the development and homeostasis of the host immune system (Troy and Kasper, 2010). Coincidentally, the delayed Bacteroidetes colonization and reduced type 1 of helper T lymphocyte (Th1) responses were illustrated in infants delivered by CS (Jakobsson et al., 2014). Furthermore, *Bacteroides* has been reported as a major component of gut microbiota in adults (Eckburg et al., 2005). In this regard, the presence of the maternal transmission in gut microbiota during delivery might contribute to the high abundance of *Bacteroides* in infants born vaginally (Vaishampayan et al., 2010; Lee et al., 2016). This indicated the essential role of maternal transmission during vaginal delivery in the establishment of early gut microbiota in infants.

Most interestingly, we found that the F/B ratio was higher in the CS group than in the VD group from day 5 until month 3. The F/B ratio was widely accepted to have an important influence in maintaining normal intestinal homeostasis. Increased F/B ratio was regarded as dysbiosis, and was usually observed with obesity (Stojanov et al., 2020). A previous systematic review and meta-analysis revealed that children born by CS were at higher risk of developing obesity in childhood, despite that findings were limited by a moderate heterogeneity among studies and the potential for residual confounding and publication bias (Kuhle et al., 2015). Moreover, a recent study from Mexico showed that infants born by CS had a strikingly high F/B ratio (Murata et al., 2020). High F/B ratio in CS delivery infants may help explain why Mexico that had one of the highest global average annual rate increases in CS led the world in childhood obesity, which may be associated with the high F/B ratio in CS delivery infants. Also, another recent study from Mexico demonstrated that Firmicutes and Bacteroidetes phyla were positively and negatively associated with obesity, respectively (Vazquez-Moreno et al., 2020). By intensively monitoring the F/B ratio at 9 time points, the CS group had a permanently higher F/B ratio than the VD group that might provide crucial evidence for the causality of high F/B ratio in the first 3 months of life and the obesity in the later life. As one study has demonstrated that mice in the CS group gained more body mass after weaning than those in the VD group and established a causal relationship between CS and increased body weight (Martinez et al., 2017). Despite CS delivery infants in our study seems to have a similar body weight with VD delivery infants at the short-term observation, it is necessary to implement long-term follow-up and determine the relationship between higher F/B ratio of early life and the risk for obesity of later life. In parallel, our results also provide new insight into the prevention and management of obesity through early regulation of the normal F/B ratio by probiotics usage or other treatments, such as fecal bacteria transplantation (Korpela et al., 2020). However, some studies revealed no significant differences in phyla abundances or F/B ratios observed between normal-weight and obese children (López-Contreras et al., 2018; Chen et al., 2020). Therefore, more studies are required to confirm the findings.

In addition, in agreement with several studies performed before (Liu et al., 2015; Lee et al., 2016; Hill et al., 2017), we observed a significantly higher level of *Clostridium* in the CS group, which has been reported to be ascribed to nosocomial infection (Penders et al., 2006). What is more, the correlation between the colonization of *Clostridium* in the gut and the increased risks of wheeze and eczema has been proven (van Nimwegen et al., 2011). CS delivery also elevated the abundances of potential pathogenic and proinflammatory bacteria associated with the hospital environment including *Klebsiella*, *Enterococcus*, and *Enterobacter* in infants, which were in accordance with previous studies (Liu et al., 2015; Reyman et al., 2019; Shao et al., 2019; Yang R. et al., 2019). And the increased proportion of *Klebsiella* in early life has been validated to be connected with later development of allergy in children (Low et al., 2017). Also, *Klebsiella*, as a common opportunistic pathogenic bacterium in hospitals, is usually associated with nosocomial infection and acts as a reservoir for a myriad of antimicrobial resistance genes (Rosenthal et al., 2012; Gibson et al., 2016), which might suggest the possible correlation between delivery mode and the increased risk of antibiotic-resistant infections (Reyman et al., 2019). In our study, *Streptococcus*, as a member of maternal skin bacteria, was more often colonized in CS-delivered infants, as other studies reported (Bäckhed et al., 2015; Moore and Townsend, 2019), while *Escherichia*–*Shigella* has also been reported to be more common in VD infants before (Bäckhed et al., 2015; Yang R. et al., 2019). Besides, *Parabacteroides* was significantly overrepresented in VD infants, which was also discovered in previous findings (Bäckhed et al., 2015; Hill et al., 2017). The lower relative abundance of *Parabacteroides* was found to be associated with a high risk for eczema in infants (Wopereis et al., 2018). Nevertheless, infants born by CS showed significantly higher relative levels of *Veillonella* in our results, which was consistent with other reports (Hesla et al., 2014; Bäckhed et al., 2015). Moreover, *Veillonella* was ascertained to be relevant to the penetrating complications of Crohn’s disease in children (Kugathasan et al., 2017). Based on the above mentioned, we conclude that gut microbial dysbiosis and delayed maturation are correlated with CS.

Our functional prediction revealed that delivery mode might have a certain impact on microbial functions. It has also been previously reported that the functional genes of taurine and hypotaurine metabolism were significantly higher in the VD group, while PTS was enriched in the CS group (Yang B. et al., 2019). Taurine was bound to bile acid to form taurocholic acid, which was involved in the fat emulsification process, for promoting the digestion of mother’s milk fat, while PTS and starch and sucrose metabolism were involved in the digestion and absorption of carbohydrates. This indicated that the gut microbiota in different delivery groups might associate with the metabolism of diverse nutrients.

The limits of this study were the small sample size and short follow-up period. Moreover, we did not collect all the fecal samples at different time points because of no defecation of infants. In spite of this, we took intensive sampling within the first 3 months of life, and the total 9 time points provided the dynamic evaluation of the early gut microflora during this early life period. Our results decipher the crucial role of delivery mode in shaping the early gut microbiota of infants.

In conclusion, CS delivery mode significantly perturbs the early colonization and development of gut microbiota. Furthermore, CS causes significantly higher F/B ratio in the first 3 months of life, which may be associated with obesity in later life. Importantly, our findings establish new evidence that CS affected the composition and development of gut microbiota in the first 3 months and provide novel insight into strategies for CS-related disorders in later life.

## Data Availability Statement

The datasets presented in this study can be found in online repositories. The names of the repository/repositories and accession number(s) can be found below: SRA, SRP283175.

## Ethics Statement

The studies involving human participants were reviewed and approved by Ethics Committee of the Children’s Hospital of Zhejiang University School of Medicine. Written informed consent to participate in this study was provided by the participants’ legal guardian/next of kin. Written informed consent was obtained from the individual(s), and minor(s)’ legal guardian/next of kin, for the publication of any potentially identifiable images or data included in this article.

## Author Contributions

GL and MJ designed the present study and drafted the manuscript. GL, YH, XS, and WZ performed the literature search. GL, ET, and BC collected the samples and performed the analyses. GL, ET, and MJ edited the manuscript. All authors have read and approved the manuscript.

## Conflict of Interest

The authors declare that the research was conducted in the absence of any commercial or financial relationships that could be construed as a potential conflict of interest.

## Publisher’s Note

All claims expressed in this article are solely those of the authors and do not necessarily represent those of their affiliated organizations, or those of the publisher, the editors and the reviewers. Any product that may be evaluated in this article, or claim that may be made by its manufacturer, is not guaranteed or endorsed by the publisher.

## Footnotes

1
http://drive5.com/uparse/

2
http://rdp.cme.msu.edu/
