# Supplementary material for: The Influence of Cesarean Section on the Composition and Development of Gut Microbiota During the First 3 Months of Life
Source: Front Microbiol. 2021 Aug 18;12:691312. doi: 10.3389/fmicb.2021.691312 (PMC8416498; doi:10.3389/fmicb.2021.691312)
Supplement: Supplementary file 1 [file Data_Sheet_1.ZIP › Supplementary Material-new revised version.doc]

***Supplementary Material***

## 1 Supplementary Figures

**Supplementary Figure 1.** The rarefaction curves of microbial diversity.

**Supplementary Figure 2.** Comparison of microbial functions between two [delivery mode](http://www.youdao.com/w/delivery mode/" \l "keyfrom=E2Ctranslation) groups (Day5, Day8, Day11). **P* <0.05; ***P* <0.01 (Wilcoxon rank-sum test).

**Supplementary Figure 3.** Comparison of microbial functions between two [delivery mode](http://www.youdao.com/w/delivery mode/" \l "keyfrom=E2Ctranslation) groups (Week2, Week4, Week6). **P* <0.05; ***P* <0.01 (Wilcoxon rank-sum test).

**Supplementary Figure 4.** Comparison of microbial functions between two [delivery mode](http://www.youdao.com/w/delivery mode/" \l "keyfrom=E2Ctranslation) groups (Week7, Month2, Month3). **P* <0.05; ***P* <0.01 (Wilcoxon rank-sum test).

## 2 Supplementary **Table**s

**Supplementary Table 1.** Generalized Linear Models results of abundance in single phylum level and days after birth for two delivery mode infants.

|  | Phylum | Day | B | Std. Error | 95% Wald Confidence Interval | | Hypothesis Test | | |
| --- | --- | --- | --- | --- | --- | --- | --- | --- | --- |
|  |  |  |  |  | Lower | Upper | Wald Chi-Square | df | Sig. |
| CS Group | Firmicutes | day5-month3 | -0.004 | 0.0009 | -0.006 | -0.002 | 22.506 | 1 | **0.000** |
|  | Proteobacteria | day5-month3 | 0.003 | 0.0009 | 0.001 | 0.004 | 7.649 | 1 | **0.006** |
|  | Bacteroidetes | day5-month3 | 0.001 | 0.0010 | -0.001 | 0.003 | 2.071 | 1 | 0.150 |
| VD Group | Firmicutes | day5-month3 | -0.002 | 0.0007 | -0.003 | 0.000 | 5.363 | 1 | **0.021** |
|  | Proteobacteria | day5-week4 | -0.012 | 0.0084 | -0.029 | 0.004 | 2.160 | 1 | 0.142 |
|  | Bacteroidetes | day5-month3 | 0.002 | 0.0011 | 0.000 | 0.004 | 3.279 | 1 | 0.070 |

**Supplementary Table 2.** Generalized Linear Models results of abundance in single genus level and days after birth for two delivery mode infants.

|  | Genus | Day | B | Std. Error | 95% Wald Confidence Interval | | Hypothesis Test | | |
| --- | --- | --- | --- | --- | --- | --- | --- | --- | --- |
|  |  |  |  |  | Lower | Upper | Wald Chi-Square | df | Sig. |
| CS Group | *Bacteroides* | day5-month3 | 0.001 | 0.0010 | -0.001 | 0.003 | 1.912 | 1 | 0.167 |
|  | *Escherichia-Shigella* | day5-month3 | 0.002 | 0.0006 | 0.001 | 0.003 | 11.163 | 1 | **0.001** |
|  | *Enterococcus* | day5-month3 | 0.000 | 0.0002 | -0.001 | 1.944E-5 | 3.439 | 1 | 0.064 |
|  | *Clostridioides* | day5-month3 | -0.002 | 0.0006 | -0.003 | 0.000 | 7.471 | 1 | **0.006** |
|  | *Clostridium_sensu_stricto_1* | day5-month3 | -0.001 | 0.0012 | -0.004 | 0.001 | 1.222 | 1 | 0.269 |
|  | *Streptococcus* | day5-month3 | -0.001 | 0.0003 | -0.001 | 0.000 | 10.783 | 1 | **0.001** |
| VD Group | *Bacteroides* | day5-month3 | 0.002 | 0.0010 | 0.000 | 0.004 | 3.265 | 1 | 0.071 |
|  | *Clostridium_sensu_stricto_1* | day8-month3 | 0.000 | 0.0002 | -0.001 | 0.000 | 1.772 | 1 | 0.183 |
|  | *Streptococcus* | day8-month3 | -0.002 | 0.0009 | -0.003 | 7.077E-5 | 3.540 | 1 | 0.060 |
|  | *Staphylococcus* | day8-month3 | 0.000 | 6.2418E-5 | 0.000 | -6.077E-6 | 4.233 | 1 | 0.040 |
